# SCG3 Protein Expression in Glioma Associates With less Malignancy and Favorable Clinical Outcomes

**DOI:** 10.3389/pore.2021.594931

**Published:** 2021-02-26

**Authors:** Yi Wang, Nan Ji, Junmei Wang, Jingli Cao, Deling Li, Yang Zhang, Liwei Zhang

**Affiliations:** ^1^ Department of Neurosurgery/China National Clinical Research Center for Neurological Diseases, Beijing Tiantan Hospital, Capital Medical University, Beijing, China; ^2^ Beijing Advanced Innovation Center for Big Data-Based Precision Medicine, Beihang University, Beijing, China; ^3^ Department of Pathology, Beijing Neurosurgical Institute, Capital Medical University, Beijing, China; ^4^ Beijing Key Laboratory of Translational Medicine for Cerebrovascular Disease, Beijing Tiantan Hospital, Capital Medical University, Beijing, China; ^5^ Beijing Neurosurgical Institute, Beijing, China

**Keywords:** SCG3, glioma, clinicopathological features, prognosis

## Abstract

**Introduction:** Secretogranin III (SCG3) physiologically participates in neurotransmitter storage/transport and is widely expressed in neuroendocrine tumors. However, there is no report on SCG3 protein expression in gliomas.

**Methods:** The method of immunohistochemical staining on a glioma tissue microarray was utilized to detect SCG3 protein expression and investigate the correlations of its expression with clinicopathological and genetic features in gliomas. The RNA-seq data of SCG3 in The Cancer Genome Atlas database was exploited to explore these correlations at the transcriptional level.

**Results:** There were 57.5% (130/226) glioma cases having SCG3 cytoplasmic staining in the tissue microarray. SCG3 expression inversely correlated with malignancy grade at both transcriptional and protein levels. The highest level was observed in oligodendroglial tumors, especially in oligodendrogliomas (ODs) with IDH-mutation/1p19q-codeletion. The lowest SCG3 expression was observed in glioblastomas (GBMs), especially in the mesenchymal subtype. Nearly a half of GBM cases (44.4%, 64/144) had any discernible SCG3 staining, and were defined as SCG3-positive by the microarray study. SCG3-positive GBM cases exhibited improved overall survival as compared with the SCG3-negative cases (29.3 vs. 14.5 months; Hazard ratio, 0.364; 95% CI, 0.216–0.612; *p* < 0.001). A multivariate Cox regression analysis also revealed SCG3 positivity as an independent favorable prognosticator in GBM patients.

**Conclusion:** SCG3 protein expression inversely correlates with glioma malignancy and predicts favorable outcomes in GBM patients.

## Introduction

Glioma is a molecularly heterogeneous brain malignancy associated with distinct therapy responses and diverse clinical outcomes [1, 2]. Molecular classification optimizes treatment selection and eventually improves prognosis of glioma patients. Decades of laborious research by scientists and clinicians worldwide have resulted in identification of numerous potential molecular markers. Among them, several markers have been widely used in clinical practice to guide accurate diagnosis and precise treatment of glioma patients. Isocitrate dehydrogenase (IDH) mutations are indicative of favorable outcomes in all glioma patients [3], whereas telomerase reverse transcriptase promoter (TERTp) mutations are associated with poor prognosis in patients with glioblastoma (GBM), a grade IV glioma [4]. O6-methylguanine-DNA methyltransferase (MGMT) promoter methylation predicts good response to temozolomide (TMZ)-based chemotherapy in glioma patients [5]. Aside from genomic markers, GBM can also be classified into four transcriptional subtypes: proneural, neural, classical and mesenchymal subtype. Each subtype has unique genetic aberrations and is associated with distinct clinical outcomes [6].

Markers at protein levels, such as GFAP [7] and Ki67 [8], have long been used in the diagnosis of gliomas. Protein markers can provide another layer of classification for cancer diagnosis. Especially, the immunochemical methods, that assay protein levels in clinical specimens, are routinely and widely used in nearly all levels of hospitals across the world. Thus, protein markers have rather high clinical value and unique advantage in the era of precision medicine.

Secretogranin III (SCG3), a member of the acid-secreting protein family known as the granins, is present in the secretory granules of various endocrine cells [9]. Physiologically, SCG3 participates in secretory granule biogenesis, neurotransmitter storage and transport, and plays an important role in peptide hormone secretion [10–12]. In pathological condition, SCG3 is associated with obesity [13], depression [14], diabetes [15], atherosclerosis [16] and estrogen-induced endocrine disorders [17]. SCG3 is also expressed widely in several tumors including some neuroendocrine tumors [18] and prostate cancer [19]. SCG3 promotes proliferation of hepatocellular carcinoma cells [20] and its transcript in peripheral blood predicts worse prognosis for REST-deficient small cell lung cancer [21]. In glioma, SCG3 has been recognized as a signature gene for the proneural subtype of GBMs [6, 22], and as a predictor for favorable prognosis in GBM patients [23, 24]. However, all these results were based on transcriptomic studies, while lacking the evidence at protein level. Herein, we examined SCG3 protein expression in different types of gliomas, and investigated the correlation of its expression with clinicopathologic characteristics as well as genetic features in gliomas. Our results show that SCG3 is a potential protein marker, that will facilitate glioma precise diagnosis.

## Materials and Methods

### Patients

A total of 267 primary glioma specimens were obtained from patients who underwent first microsurgical resection in Beijing Tiantan Hospital, Capital Medical University from 2011 to 2017. These specimens (one tumor core from each specimen) were used to construct a tissue microarray. Among them, 226 cases of specimens got successful SCG3 quantification and were evaluated in this study (Supplementary Table S1). This study was supported by the Neurosurgical Clinical Information and Biobanking Project of Beijing Tiantan Hospital (Brain Tumor Section) and was approved by the ethics committee of Beijing Tiantan Hospital (KY2014-021-02). The clinicopathologic characteristics and genetic features, related to these specimens, were extracted from the clinical information database. RNA-seq data pertaining to SCG3 in gliomas (160 GBMs and 515 Grade II/III gliomas) and the associated clinical and molecular information were extracted from The Cancer Genome Atlas (TCGA; http://cancergenome.nih.gov/) for analysis.

### Immunohistochemistry

An immunohistochemical (IHC) method was applied to detect SCG3 protein expression in a glioma tissue microarray. Briefly, the sections of tissue microarray were deparaffinized, rehydrated and subjected to heat-induced epitope recovery, according to a routine protocol. The sections were blocked in a goat serum working solution for 1 h and then incubated with anti-SCG3 (Cat# NBP1-89825, Novus, Colorado, United States) antibodies diluted 1:200 in a solution of 0.3% PBST and 10% goat serum overnight at 4°C. After the incubation in a secondary antibody (Cat#ZB-2301, ZSGB-BIO, Beijing, China) diluted 1:1,000 in a solution of 0.3% PBST and 10% goat serum for 1 h, the sections were stained with a DAB peroxidase substrate solution for 15 min and counterstained with hematoxylin for 10 min. The sections were then dehydrated and mounted according to a standard protocol.

SCG3 protein expression was semi-quantified by the SCG3 staining extent, which was calculated as the number of nuclei of SCG3-positive cells divided by the number of all nuclei in the section. Meanwhile, we also defined the case with any discernible SCG3 staining as a positive case, and compared the prevalence of SCG3-positive cases between different types of gliomas.

### Quantitative Real-Time PCR

Among the cases included in the tissue microarray, 46 cases of frozen tumor tissues, including 25 grade II/III and 21 grade IV glioma tissues, were subject to quantitative real-time PCR (qPCR) (Supplementary Table S2). Total RNA was isolated from tissues using the TRIzol reagent (Invitrogen, Carlsbad, CA, United States) according to the protocol provided by the manufacturer. RNA was dissolved in diethylpyrocarbonate (DEPC)-treated water, and reverse transcribed using the SuperScript III First Strand Synthesis Super Mix Kit (Thermo Fisher, United States) according to the manufacturer's instruction. cDNA was quantitated with RT-qPCR using the Luna Universal qPCR Master Mix (M3003L, NEB) by the Light Cycler Instrument (BIO-RAD CFX96). The PCR program consisted of an initial denaturation at 95°C for 60 s followed by 40 cycles of 95°C for 15 s and 60°C for 30 s. Heating and cooling rates and all other parameters were at the manufacturers’ pre-set levels.

The relative quantification of the PCR products was performed after normalization against GAPDH, using the comparative cycle threshold method. The GAPDH mRNA was amplified with primers 5′-CGG​AGT​CAA​CGG​ATT​TGG​TCG​TA-3′ and 5′-AGC​CTT​CTC​CAT​GGT​GGT​GAA​GAC-3′. The specific PCR primers for SCG3 were obtained from Bio-TNT (Cat.PRIM046178) (5′-TCA​TCA​ACT​AGA​CGG​GAC​TCC-3′/5′- AAT​CTT​GTC​AAA​CAC​GGC​TCT- 3′).

### Western Blot

Among the cases included in the tissue microarray, 53 cases of frozen tumor tissues, including 28 grade II/III and 25 grade IV glioma tissues, were used to quantify SCG3 protein expression by Western blot (Supplementary Table S3). Each case was tested once. GAPDH was applied as a loading control. Proteins were extracted from glioma tissues, loaded onto SDS-PAGE gels and transferred to PVDF membranes (Cat. IPVH00010, Pore size: 0.45 µm, Merck Millipore, United States). Amount of proteins loaded for each case is 30 μg. The membranes were first blocked with 5% skim milk powder (SMP) in TBST buffer for 1 h at room temperature, then were incubated with an anti-SCG3 antibody (Cat. NBP1-89825, Novus, United States) or anti-GAPDH (Cat. G1020V, Beijing GXY Tech., China), diluted 1:2,500 or 1:5,000, respectively, with 5% SMP in TBST buffer, at 4°C overnight. After intensive wash with TBST buffer, the membranes were incubated with a horseradish peroxidase-conjugated secondary antibody (the anti-rabbit antibody for SCG3 and anti-mouse antibody for GAPDH; both were from EarthOX, United States), diluted 1:5,000 with 5% SMP in TBST buffer, for 60 min at room temperature. The membranes were developed using Immobilon Western chemiluminescent horseradish peroxidase substrate (Millipore, United States). The protein signals were detected and quantified with Chemi DOC MP system (BIORAD, United States). The relative quantification of SCG3 was performed after normalization against GAPDH.

### Statistical Analyses

All statistical analyses were performed using SPSS software version 23.0 (IBM, New York, United States). Categorical data were compared using chi-squared tests or Fisher’s exact test, and continuous data were compared using Student’s *t*-test, one-way analysis of variance or non-parametric test. Kaplan-Meier analysis was used to estimate overall survival from the date of surgery to the date of death or the last follow-up. The log-rank test was applied to estimate between-group differences and to examine the factors that impact the overall survival of patients. Multivariate Cox regression analysis was fitted to select the independent prognostic factors. A two-tailed *p*-value < 0.05 was considered significant.

## Results

### High Prevalence of SCG3 Protein Expression in Gliomas

SCG3 was first identified by our preliminary proteomic study as a protein with 2.1-fold higher expression in oligodendrogliomas than GBMs (Supplementary Figure S1). In this study, the IHC method was used to detect SCG3 protein expression on a glioma tissue microarray (TMA). The TMA was made up of 267 cases of primary glioma specimens, among which 226 cases got successful SCG3 quantification. They included 44 grade II, 38 grade III and 144 grade IV gliomas (Supplementary Table S1).

**FIGURE 1:**
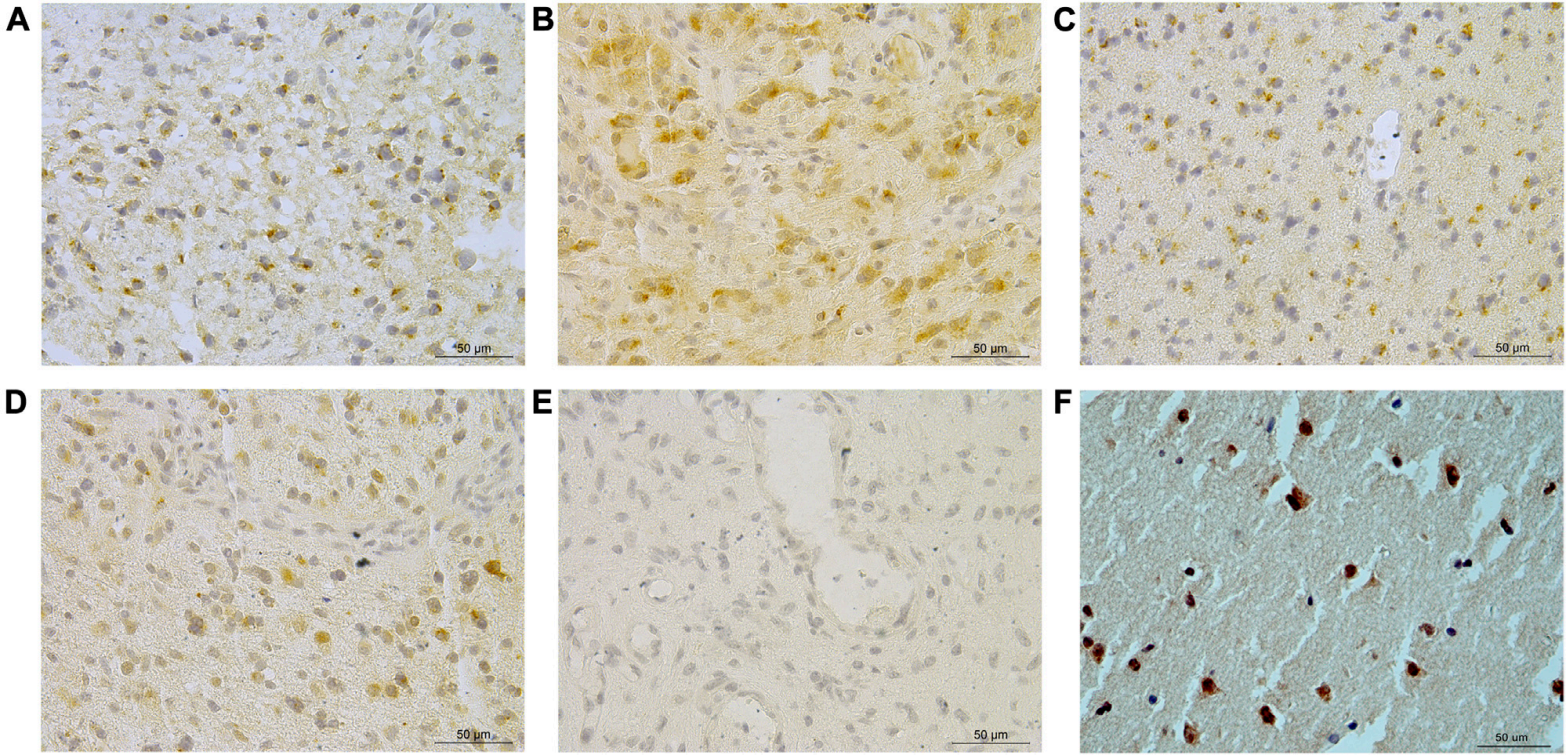
Representative immunohistochemical staining of SCG3 in gliomas. **(A–D)** Representative staining of SCG3 in grade II astrocytoma **(A)**, grade III oligodendroglioma **(B)**, grade II oligoastrocytoma **(C)** and grade IV glioblastoma **(D)**. **(E)**. Negative control. **(F)**. Representative staining of SCG3 in normal brain tissue. Scale bar 50 µm.

A total of 57.5% (130/226) glioma cases had any discernible SCG3 staining. The staining extent varied remarkably among different grades and histological diagnoses in gliomas (Figures 1A–E). The staining pattern of SCG3 was cytoplasmic in glioma cells; no vascular structural enhancement was noted throughout the tumor tissue (Figures 1A–E). The staining pattern was similar among different types of gliomas. We also stained three cases of peritumor normal brain tissues, which all showed strong cytoplasmic staining in neurons (Figure 1F). Together, our result demonstrated a wide and varying staining of SCG3 in gliomas, as well as an intensive staining in neurons of normal brain tissues.

### Inverse Correlation of SCG3 Expression With Glioma Malignancy Grades

**FIGURE 2:**
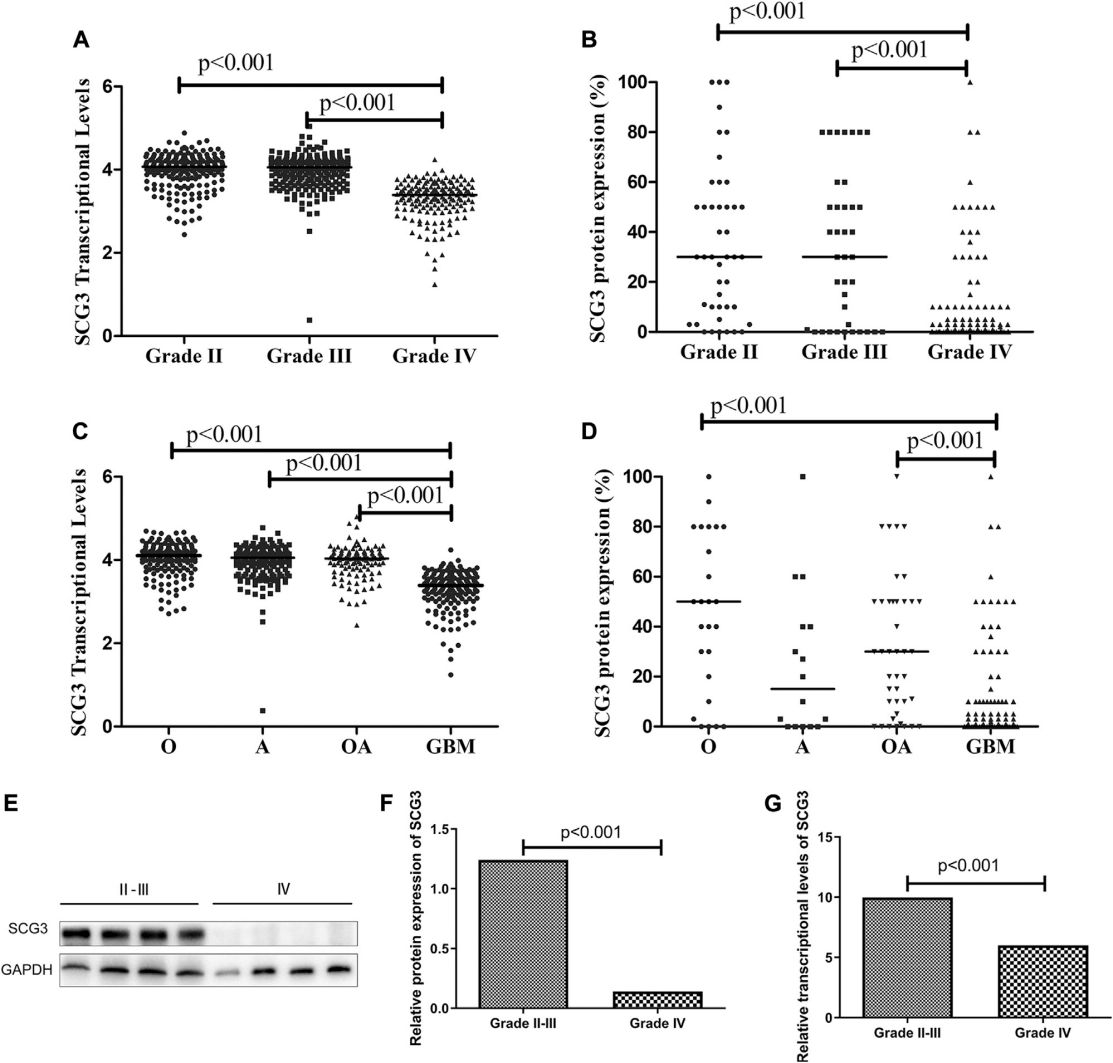
Differential SCG3 expression in gliomas with different histological grades and types. **(A–D)**. Difference of SCG3 expression in gliomas with different histological grades and types was investigated by analyzing RNA-seq data of SCG3 in TCGA database (transcriptional levels, **A**,**C**) or evaluating immunohistochemical staining extents (protein expression, **B**,**D**) on a glioma tissue microarray. **(E)**. Representative Western blot for SCG3 protein expression in grade IV and II/III gliomas. **(F–G)**. Difference of SCG3 protein expression by Western blot **(F)** and transcriptional levels by quantitative real-time PCR **(G)** between grade IV and II/III gliomas. O, Oligodendroglioma; OA, oligoastrocytoma; A, astrocytoma; GBM, glioblastoma. The horizontal line **(A–D)** or box height **(F**,**G)** represent the median value. Multiple comparison by Two-sided Kruskal-Wallis H test **(A–D)**, *p* values adjusted by Bonferroni correlation; Two-group comparison by two-sided Mann-Whitney U test **(F**,**G)**.

To investigate the correlations of SCG3 protein expression with the clinicopathological and genetic features in glioma, we semi-quantified SCG3 expression levels by the SCG3 staining extent in each spot of TMA and compared them between different types of gliomas. Meanwhile, we exploited the RNA-seq data of SCG3 in The Cancer Genome Atlas (TCGA) database to examine those correlations at the transcriptional level. As shown in Figures 2A,B, SCG3 expression was significantly lower in grade IV gliomas (GBMs) than grade II/III gliomas at both transcriptional and protein levels, whereas no difference was observed between grade II and III gliomas. Moreover, we defined the case having any discernible SCG3 staining as a SCG3-positive case and compared the proportions of SCG3-positive cases in each type of gliomas. Consistently, the positive proportion in Grade II gliomas was as high as 86.4%, followed by 73.7% in grade III glioma. Both were much higher than the 44.4% in grade IV glioma (Supplementary Table S1).

Regarding to SCG3 expression in different histological types, apart from the lowest staining extent or positive proportion observed in GBMs (Figures 2C,D; Supplementary Table S1), that was consistent with the aforementioned results (Figures 2A,B; Supplementary Table S1), oligodendroglial tumors including oligodendroglioma (O) and oligodendroastrocytoma (OA) trended to overexpress SCG3 protein relative to astrocytoma (Figure 2D; Supplementary Table S1), although the difference did not reach a statistically significant level. Therefore, SCG3 expression was inversely correlated with glioma malignancy and trended to be the highest level in oligodendroglial tumors. The results from Western blotting and quantitative real-time PCR (qPCR) also verified the inverse correlation of SCG3 with glioma malignancy grades (Figures 2E–G).

### Highest SCG3 Expression in IDH-Mutant/1p19q-Codeleted Gliomas

**FIGURE 3:**
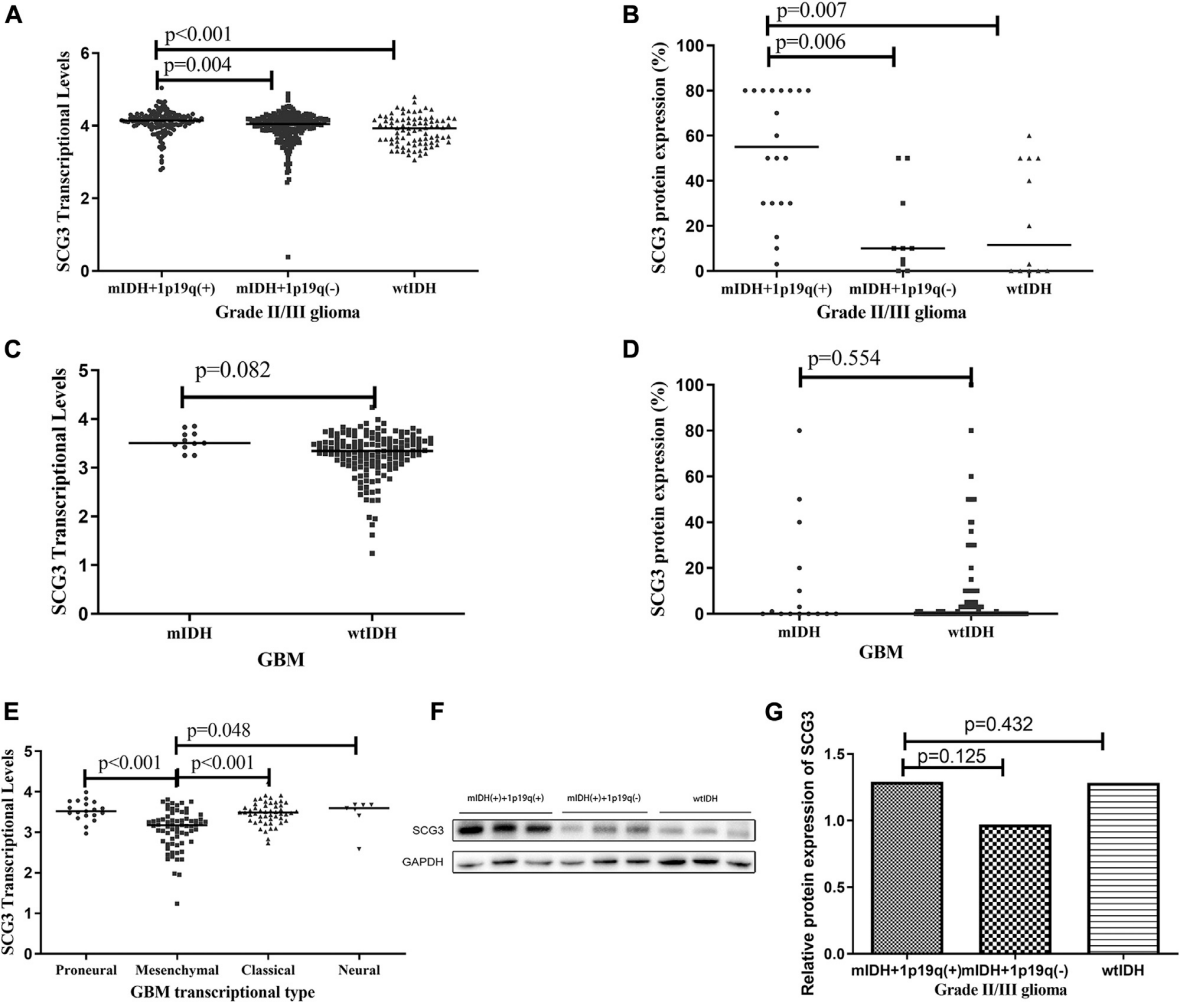
Differential SCG3 expression in gliomas with different molecular types. **(A–E**). Difference of SCG3 expression in gliomas with different molecular types was investigated by analyzing RNA-seq data of SCG3 in TCGA database (transcriptional levels; **A**,**C**,**E**) or evaluating immunohistochemical staining extents (protein expression, **B**,**D**) on a glioma tissue microarray. **(F)**. Representative Western blot for SCG3 protein expression in different molecular types of grade II/III gliomas. **(G)**. Difference of SCG3 protein expression by Western blot in different molecular types of grade II/III gliomas. mIDH+1p19q(+), wtIDH and mIDH + 1p19q(−) represent IDH-mutant/1p19q-codeleted, IDH-wildtype and IDH-mutant/non-1p19q-codeleted, respectively; mIDH and wtIDH represent IDH-mutant and IDH-wildtype, respectively. IDH mutation was defined as IDH1-R132H or IDH2-R172K. The horizontal line **(A–** **E)** or box height **(G)** represent the median value. Multiple comparison by two-sided Kruskal-Wallis H test **(A,B,E,G)**, *p* values adjusted by Bonferroni correlation; Two-group comparison by two-sided Mann-Whitney U test **(C and D)**.

According to 2016 WHO classification of gliomas [25], grade II/III gliomas can be grouped into three subtypes: IDH-wildtype, IDH-mutant/1p19q-codeleted and IDH-mutant/non-1p19q-codeleted gliomas. The IDH-mutant/1p19q-codeleted glioma is commonly recognized as genetically confirmed oligodendrogliomas (ODs) [25]. GBMs can be classified into IDH-mutant and IDH-wildtype GBMs. In this microarray, 80.1% (181/226) cases had full genetic information for molecular subtyping. Both the TCGA analysis and microarray study showed that IDH-mutant/1p19q-codeleted gliomas had the highest level of SCG3 expression among the three subtypes of grade II/III gliomas (Figures 3A,B). Consistently, the proportion of SCG3-positive cases was 100% in IDH-mutant and 1p19q-codeleted gliomas, higher than 80 or 58.3% in the other two subtypes (Supplementary Table S1). The similar trend was also observed by the Western blot study (Figures 3F,G), although it did not reach a statistically significant level, given the limited sample-size in the validation study.

SCG3 expression was similar between IDH-mutant and wildtype GBMs (Figures 3C,D). According to transcriptional signatures, GBM can also be classified into four subtypes: proneural, neural, classical and mesenchymal GBM [6]. We then compared the transcriptional levels of SCG3 among the four subtypes. As shown in Figure 3E, SCG3 transcriptional level was significantly lower in mesenchymal subtype than the others. We did not observe significant difference of SCG3 expression between gliomas grouped by other common genetic features in gliomas, such as TERT promoter mutations (Supplementary Figures S2A,B) and MGMT promoter methylation (Supplementary Figures S2C,D). Taken together, SCG3 expression was significantly higher in IDH-mutant/1p19q-codeleted gliomas than the other subtypes of glioma, and SCG3 transcriptional activity was decreased in the mesenchymal subtype of GBMs.

### Presence of SCG3 Protein Expression Indicating Favorable Outcomes in GBM Patients

**FIGURE 4:**
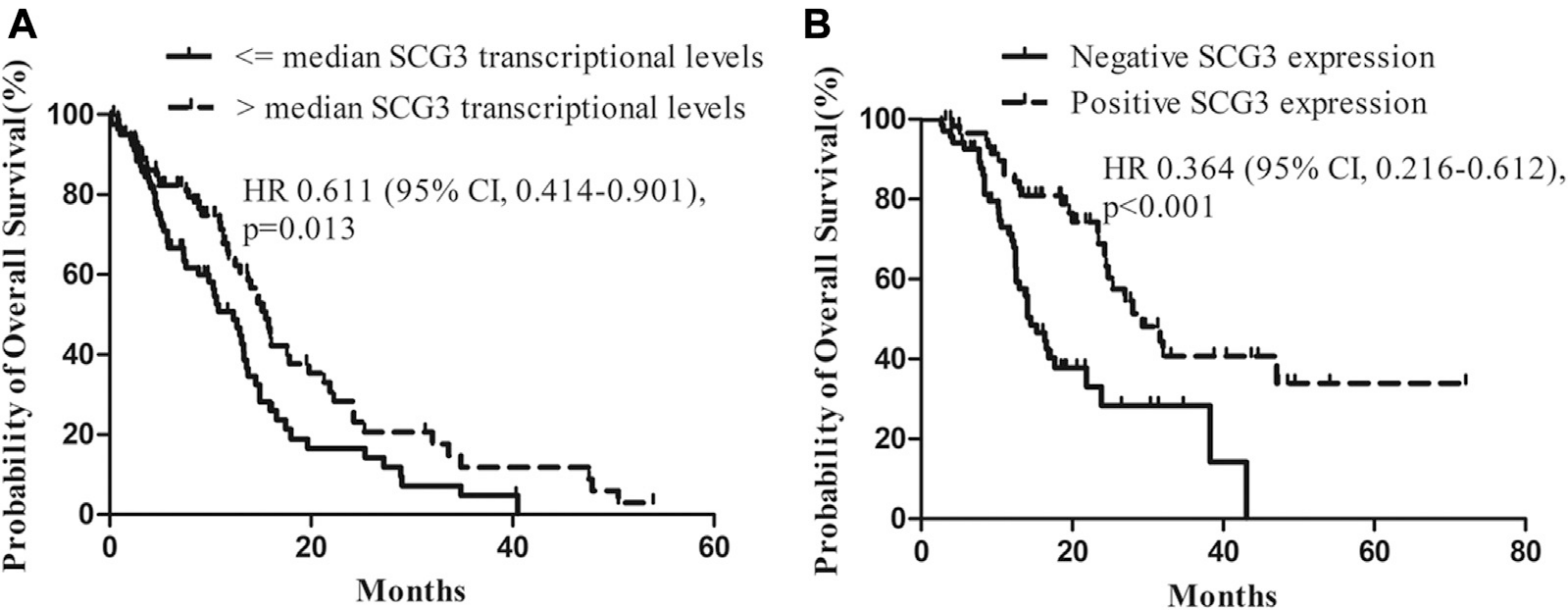
Impact of SCG3 expression on overall survival for glioblastoma (GBM) patients. **(A)**. Kaplan–Meier estimates of overall survival in GBM patients with high vs low transcriptional levels of SCG3 within the TCGA database. The median transcriptional level of SCG3 in the dataset was used as a cutoff. **(B)**. Kaplan–Meier estimates of overall survival in GBM patients with positive vs negative immunohistochemical staining of SCG3 within the glioma microarray study. Two-sided Log-rank test was applied to estimate difference.

Since SCG3 physiologically is expressed in neurons of normal brain tissues (Figure 1F) and its expression in gliomas correlated inversely with malignancy grades (Figure 2), we hypothesized that SCG3 expression would reflect a state of maturity or differentiation in gliomas, thereby would inform better clinical outcomes in glioma patients. To test it, we first examined the association of SCG3 transcriptional levels with the prognoses of GBM patients in the TCGA study. According to the transcriptional levels of SCG3, we divided these patients into two groups: above-median group (SCG3 levels >= the median value) and below-median group (SCG3 levels < the median value). In this GBM cohort, the above-median group had extended overall survival time than the below-median group (Figure 4A).

Next, we examined the value of SCG3 protein in predicting GBM patient outcomes. All the cases in the microarray were under a follow-up program at our hospital. The median overall survival is 25.3 months for grade II/III gliomas and 16.1 months for GBMs (Supplementary Table S1). Concerning that nearly a half (44.4%) of GBMs was SCG3-positive, i.e., having any discernible SCG3 staining, we categorized GBM cases into the SCG3-positive and SCG3-negative group for survival analysis. As a result, median overall survival for the SCG3-positive patients was 29.3 months as compared with 14.5 months in the SCG3-negative patients (hazard ratio, 0.364; 95% CI, 0.216–0.612; *p* < 0.001) (Figure 4B). Furthermore, apart from some well-known favorable prognosticators including high preoperative KPS (Karnofsky Performance Status, hazard ratio, 0.498; 95% CI, 0.282–0.878; *p* = 0.016) and receiving chemoradiotherapy (hazard ratio, 0.434; 95% CI, 0.257–0.732; *p* = 0.002), SCG3 positivity was also shown by a multivariate Cox regression model as another independent predictor for better outcomes in this cohort of GBM patients (Table 1). However, neither the TCGA analysis nor microarray study showed a correlation of SCG3 expression with prognosis for grade II/III glioma patients (Supplementary Figure S3). Together, our result demonstrates that presence of SCG3 protein expression independently predicted favorable prognosis in GBM patients, indicating the potential of SCG3 as a protein prognostic biomarker of gliomas.

**TABLE 1:** A multivariate Cox regression model predicting median overall survival in glioblastoma patients.

Variables		Multivariate analysis		
		Hazard ratio	95% CI	*p* value
Age	Per 1-year increment	1.014	0.989–1.040	0.269
IDH	Mutant vs. wildtype	0.647	0.257–1.768	0.423
Preoperative KPS	>70 vs. ≤ 70	0.498	0.282–0.878	0.016
Chemoradiotherapy	With vs. without	0.434	0.257–0.732	0.002
SCG3	Present vs. absent	0.382	0.221–0.662	0.001

IDH, isocitrate dehydrogenase; KPS, karnofsky performance status.

## Discussion

SCG3 belongs to the granin family, which includes chromogranin A, chromogranin B and SCG2-7 [10]. SCG3 participates in pathogenesis of hepatocellular carcinoma and is widely expressed in various neuroendocrine tumors [18, 20]. Recently, several studies have identified high SCG3 transcriptional activity as a signature for the proneural type of GBMs [6, 22]. This is the first study reporting SCG3 protein expression in gliomas. SCG3 protein was detected in more than a half (57.5%) of gliomas by the IHC method, indicating high prevalence of SCG3 protein expression in gliomas. We also observed that SCG3 expression varied significantly among different types of gliomas, especially it inversely correlated with glioma malignancy grades (Figure 2). Given that SCG3 functions in mature neurons (Figure 1F) [26] and it down-regulates when glioma cells are in a dedifferentiated state [27], this finding implicates SCG3 protein expression as an indicator of differentiated state for glioma diagnosis.

Mitsuaki Shirahata et.al, has observed higher transcriptional activity of SCG3 in anaplastic ODs than GBMs in 2007 [28]. Our preliminary proteomic study confirmed the above correlation at protein level (Supplementary Figure S1). In this study, we uncovered IDH-mutant/1p19q-codeleted ODs not only exhibited 100% prevalence of SCG3 protein expression (Supplementary Table S1), but also expressed the highest levels among all types of malignant gliomas (Figure 3B). This finding reflects that IDH-mutant/1p19q-codeleted ODs would be the most differentiated type of malignant gliomas, corroborating the fact they exhibit the most favorable prognosis in malignant gliomas [29]. Additionally, concerning to the extremely high prevalence of SCG3 expression in ODs, whether SCG3 engaging pathogenesis of this type of glioma requires further mechanism studies in the future.

Mounting evidences have already established a link of high SCG3 transcriptional levels with improved outcomes in GBM patients [23, 24]. Our microarray study expanded it to the protein level (Figure 4). SCG3 protein expression was present in around a half (44.4%) of GBM cases (Supplementary Table S1). These cases had significantly extended survival time as compared with those without SCG3 expression (Figure 4B). Moreover, SCG3 positivity independently predicted favorable survival in GBM patients (Table 1). The intrinsic mechanism for the association remains unknown. However, two factors may be implicated in this link. First, SCG3 is a signature gene for the proneural subtype that exhibits more favorable outcome in comparison to the other subtypes of GBMs [6, 22]. In this study, we did not observe proneural GBMs harboring the highest transcriptional level of SCG3, but we observed the mesenchymal subtype exhibiting the lowest expression (Figure 3E). The mesenchymal subtype linked to the poorest outcome among the four subtypes of GBM [30]. As such, SCG3 protein expression would indicate more likely as proneural subtype or less likely as mesenchymal subtype, both leading to its link to the improved outcomes. Second, higher SCG3 expression may refer to more differentiated state, which may also contribute to the extended overall survival time.

In this study, we uncovered the value of SCG3 as a protein marker for glioma diagnosis and prognostication. Thus, IHC-based SCG3 protein detection can be integrated into other diagnostic modalities for more precise diagnosis in glioma patients. Although this study is not small in sample size, including approximately 230 glioma patients, it is still a retrospective study. The real-world value of SCG3 as a protein marker in clinical application should be determined in a larger population study with prospective design.

## Conclusion

SCG3 protein expression correlates inversely with glioma malignancy and predicts favorable clinical outcomes in GBM patients.

## Data Availability

The raw data supporting the conclusions of this article will be made available by the authors, without undue reservation.

## Abbreviations

SCG3, secretogranin III; TCGA, The cancer genome Atlas; GBMs, glioblastomas; LGGs, lower grader gliomas; ODs, oligodendrogliomas; OAs, oligoastrocytomas; IHC, immunohistochemical staining; MGMT, O6-methylguanine-DNA methyltransferase; IDH, isocitrate dehydrogenase; TERTp, telomerase reverse transcriptase promoter; TMZ, temozolomide; FFPE, formalin-fixed and paraffin-embedded; TMA, tissue microarray
