# Supplementary material for: SCG3 Protein Expression in Glioma Associates With less Malignancy and Favorable Clinical Outcomes
Source: Pathol Oncol Res. 2021 Feb 26;27:594931. doi: 10.3389/pore.2021.594931 (PMC8262226; doi:10.3389/pore.2021.594931)

**Supplemental Figure 1: Identification of SCG3 protein overexpression in oligodendrogliomas by a quantitative proteomic methods.**

An iTRAQ-based quantitative proteomic method was applied to identify proteins that were differentially expressed between oligodendrogliomas (OGs) and glioblastomas (GBMs). SCG3 was identified as a protein overexpressed in OGs relative to GBMs. The quantitative information of 3 unique peptides of SCG3 was shown below (iTRAQ-114: grade 2 OGs, iTRAQ-115: grade 3 OGs and iTRAQ-116: GBMs). As a result, the expression of protein SCG3 in grade 2 or 3 OGs increased by 2.1-fold compared to GBMs.

1. **Peptide 1: EANNYEEDPNKPTSWTENQAGK**


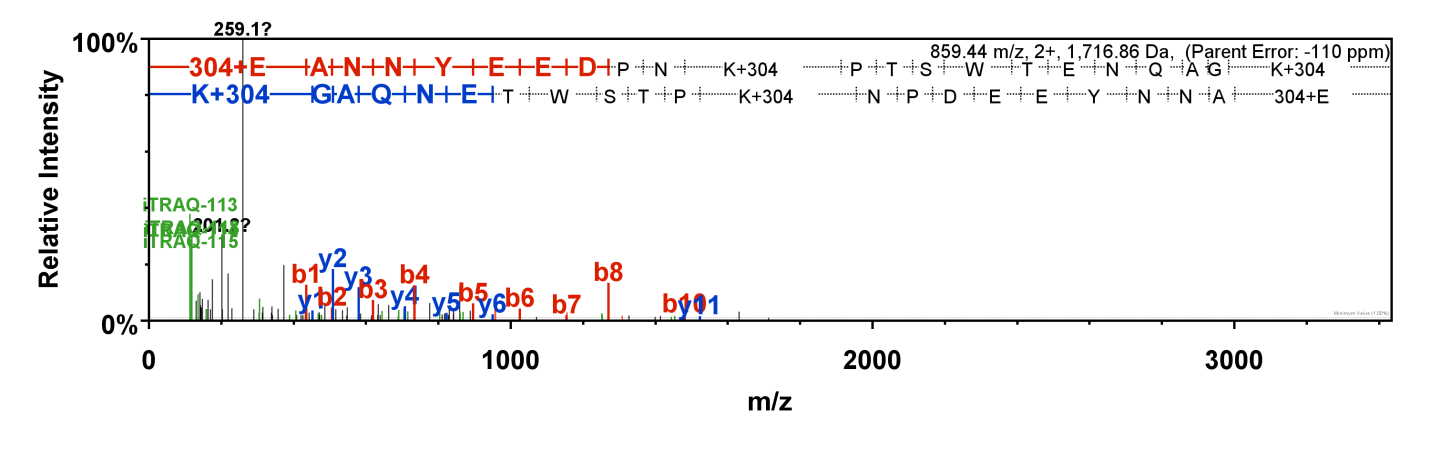


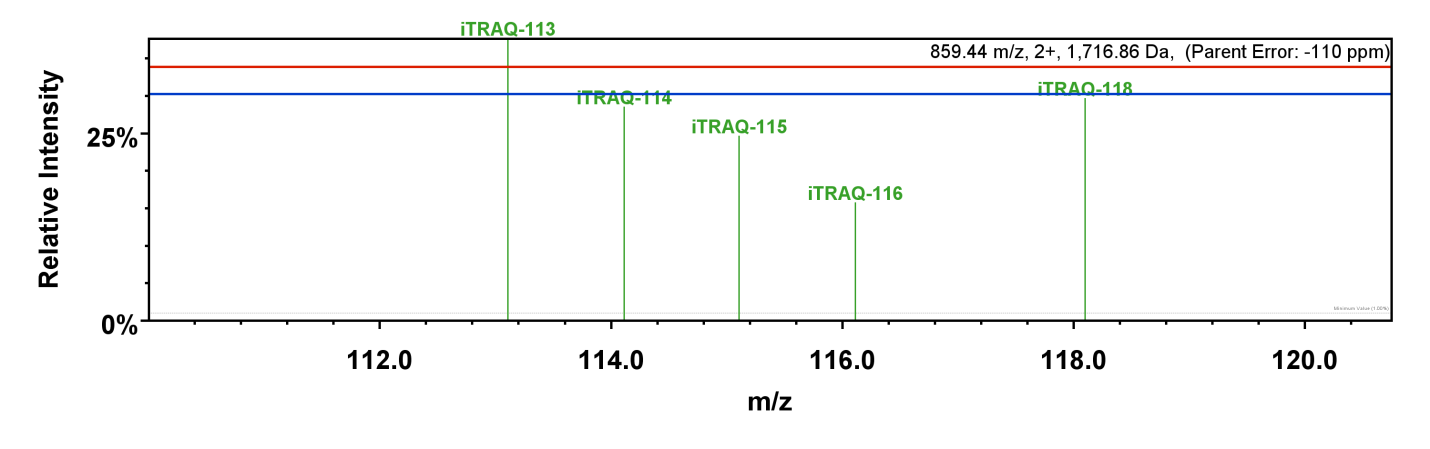


**2: Peptide 2: LNVEDVDSTK**


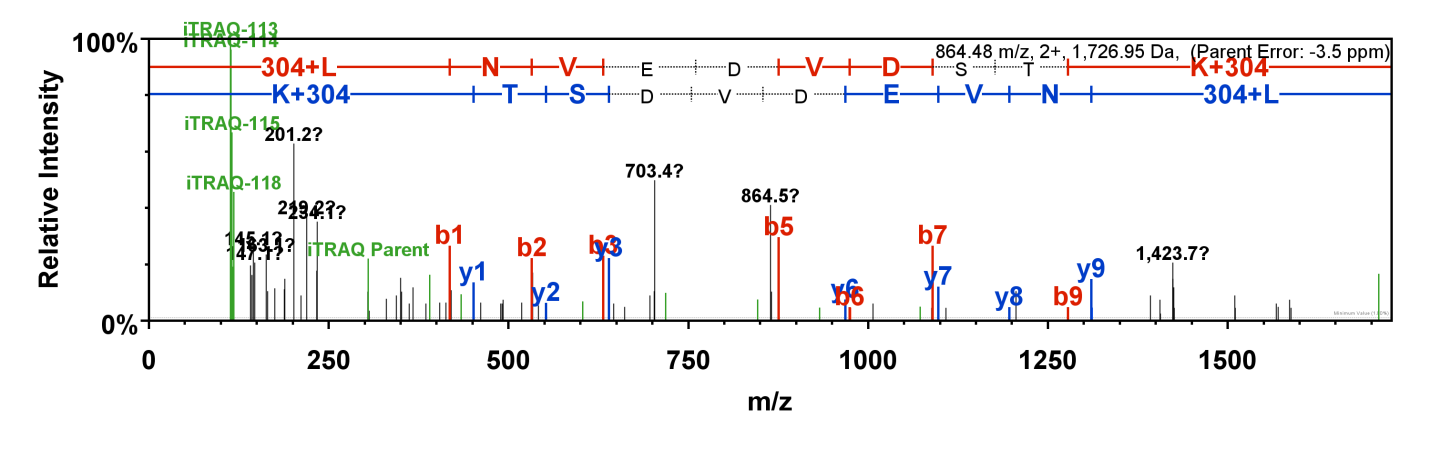


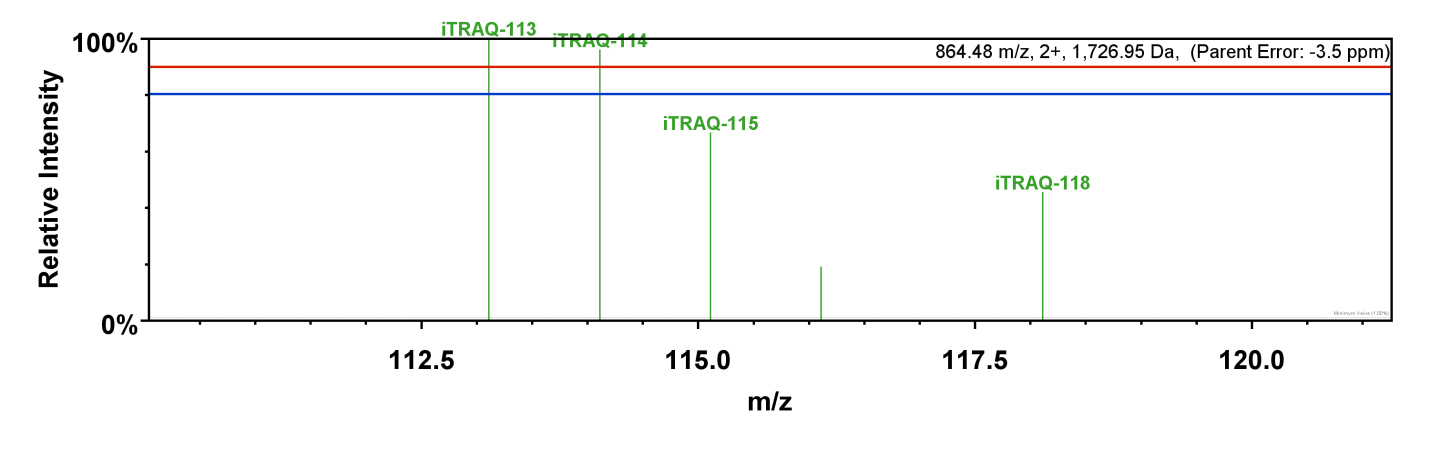


**3: Peptide 3: TEAYLEAIR**


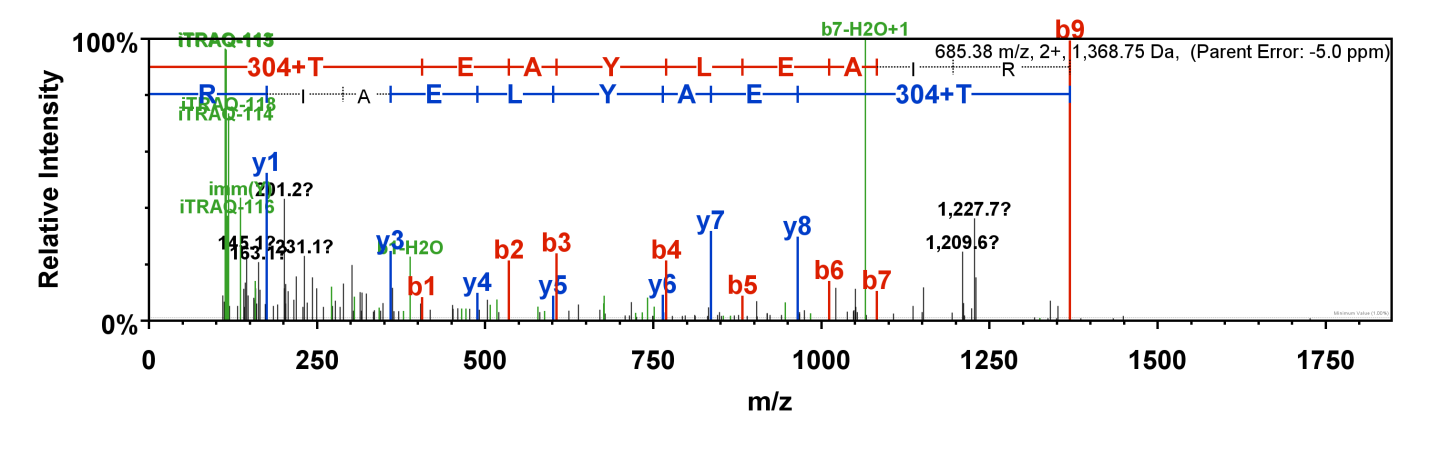


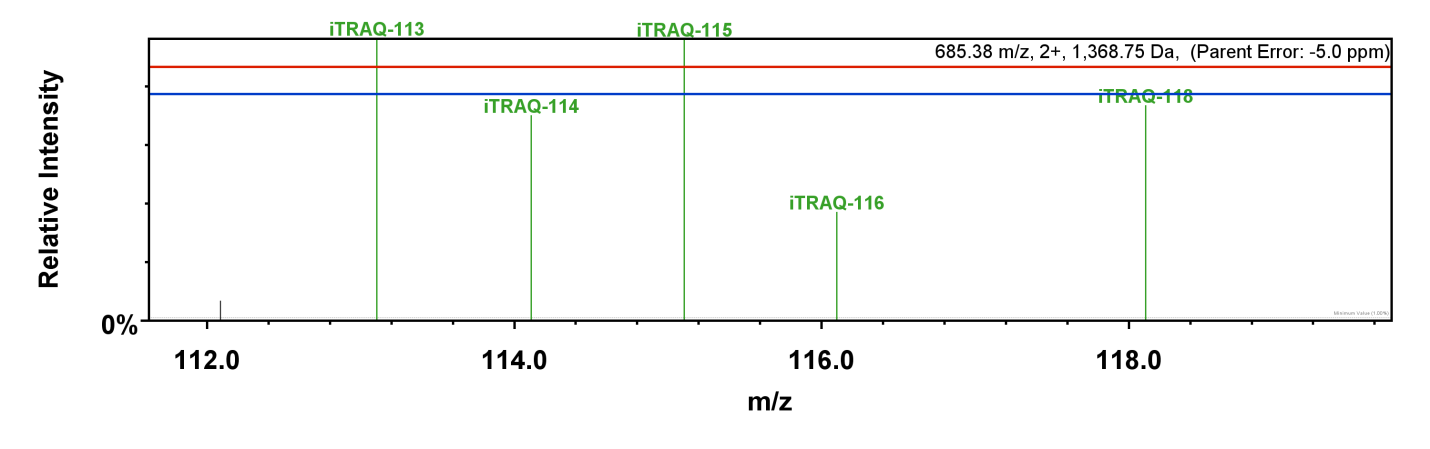

Supplement: Supplementary file 1 [file Table1.DOCX]
