# Supplementary material for: SCG3 Protein Expression in Glioma Associates With less Malignancy and Favorable Clinical Outcomes
Source: Pathol Oncol Res. 2021 Feb 26;27:594931. doi: 10.3389/pore.2021.594931 (PMC8262226; doi:10.3389/pore.2021.594931)

**Supplemental Figure 2. SCG3 expression difference between gliomas grouped by TERT promoter mutations or MGMT promoter methylation.**

The difference of SCG3 expression in gliomas with different molecular types was investigated by analyzing RNA-seq data of SCG3 in TCGA database (transcriptional levels; a & c) or evaluating immunohistochemical staining extents of SCG3 on a glioma tissue microarray (protein expression; b & d). a. Transcriptional levels of SCG3 between mTERT (mutant TERT promoter) and wtTERT (wildtype TERT promoter) gliomas. b. Protein expression of SCG3 between mTERT and wtTERT gliomas. c. Transcriptional levels of SCG3 between meMGMT (methylated MGMT promoter) and unmeMGMT (methylated MGMT promoter) gliomas. d. Protein expression of SCG3 between meMGMT and unmeMGMT gliomas. Two-sided Mann-Whitney U test. TERT promoter mutation was defined as TERT-C250T or TERT-C228T.


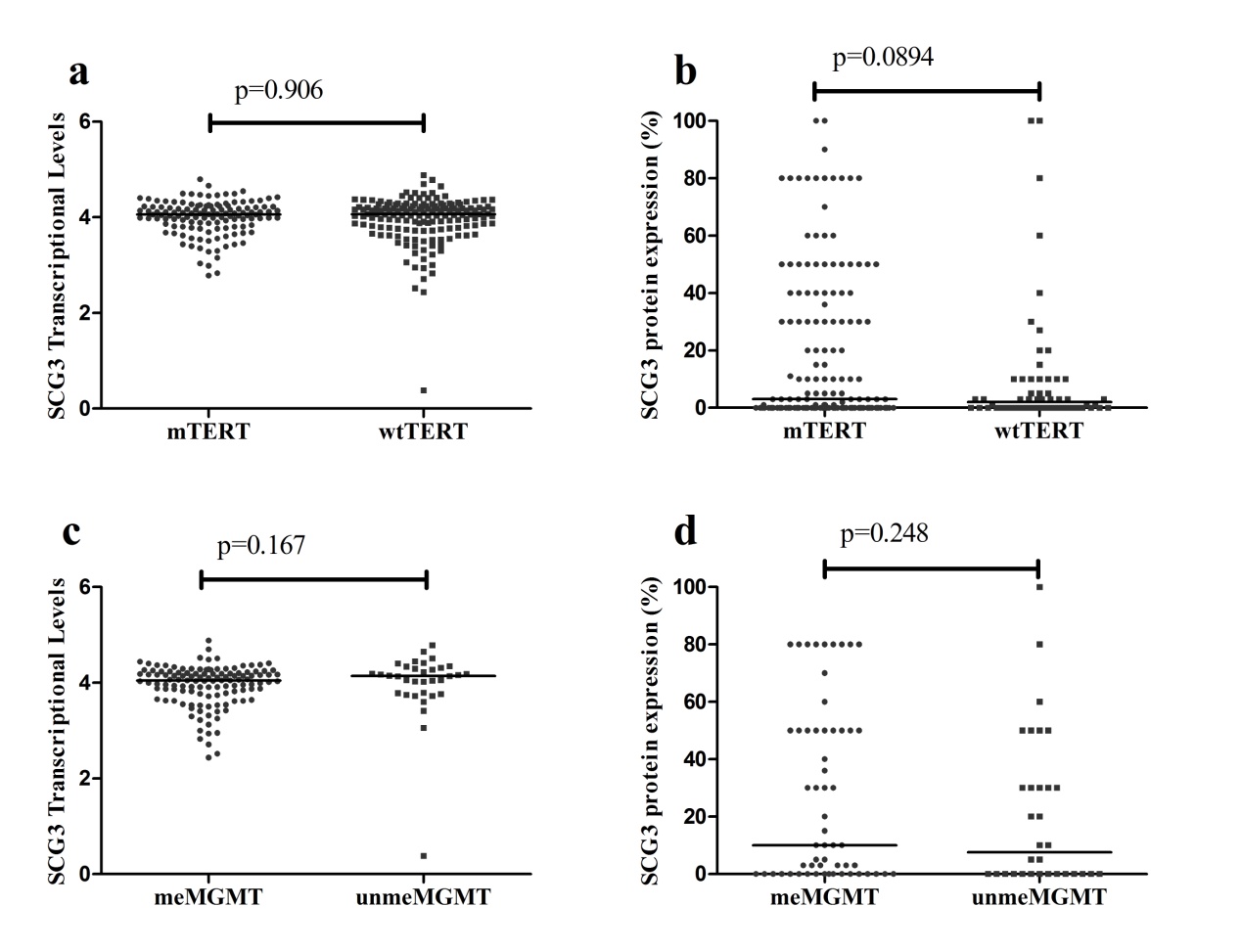

Supplement: Supplementary file 2 [file Table2.DOCX]
