# Supplementary material for: SCG3 Protein Expression in Glioma Associates With less Malignancy and Favorable Clinical Outcomes
Source: Pathol Oncol Res. 2021 Feb 26;27:594931. doi: 10.3389/pore.2021.594931 (PMC8262226; doi:10.3389/pore.2021.594931)

**Supplemental Figure 3.** **Impact of SCG3 expression on overall survival for grade II/III patients**

a. Kaplan–Meier survival estimates of overall survival in grade II/III glioma patients with high vs low transcriptional levels of SCG3 within the TCGA database. The median transcriptional level of SCG3 in the dataset was used as a cutoff. b. Kaplan–Meier survival estimates of overall survival in grade II/III glioma patients with high vs low protein expression of SCG3 within the glioma microarray study. The median protein expression of SCG3 in the dataset was used as a cutoff. Two-sided Log-rank test was applied to estimate difference.


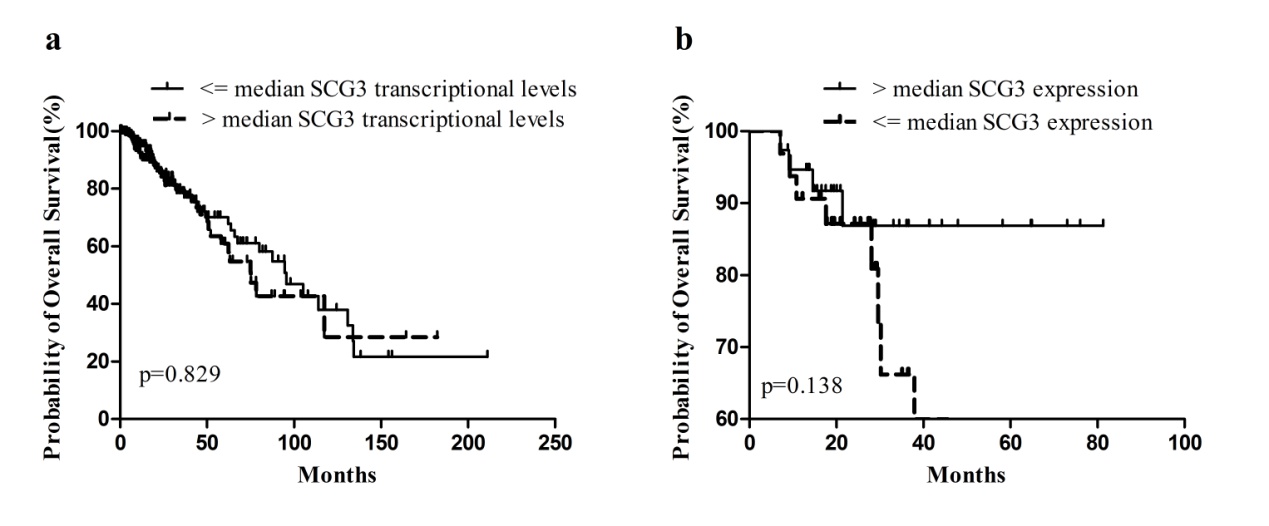

Supplement: Supplementary file 3 [file Table3.DOCX]
