# Supplementary material for: SCG3 Protein Expression in Glioma Associates With less Malignancy and Favorable Clinical Outcomes
Source: Pathol Oncol Res. 2021 Feb 26;27:594931. doi: 10.3389/pore.2021.594931 (PMC8262226; doi:10.3389/pore.2021.594931)
Supplement: Supplementary file 4 [file Table4.DOCX]

**Supplemental Table 1: Clinical characteristics of the patients in the tissue microarray study.**

| **Variables** | **Number of cases with positive SCG3 staining** | | **Number of cases with negative SCG3 staining** | **Positive proportion**  **in the IHC study, %** | ***p* value^*^** |
| --- | --- | --- | --- | --- | --- |
| **Gender:** |  | |  |  |  |
| Male | 84 | | 69 | 54.9 | 0.25 |
| Female | 46 | | 27 | 63.0 |  |
| **Tumor locations:** |  | | | |  |
| FPTO | 119 | | 89 | 57.2 | 0.72 |
| Middle-line | 7 | | 4 | 63.6 |  |
| Multiple | 3 | | 1 | 75.0 |  |
| Unknown | 1 | | 2 | 33.3 |  |
| **Pathological grade (WHO):** |  | | | |  |
| II | 38 | | 6 | 86.4 |  |
| III | 28 | | 10 | 73.7 | <0.0001 |
| IV | 64 | | 80 | 44.4 |  |
| **Histopathology:** |  | |  |  |  |
| Astrocytoma | 11 | | 5 | 68.8 | <0.0001 |
| Oligodendroglioma | 21 | | 4 | 84.0 |  |
| Oligoastrocytoma | 34 | | 7 | 82.9 |  |
| GBM | 64 | | 80 | 44.4 |  |
| **Molecular Subtype:** |  | |  |  |  |
| IDH-wildtype LGG | 7 | | 5 | 58.3 | 0.009 |
| IDH-mutant &1p19q co-deleted LGG | 20 | | 0 | 100.0 |  |
| IDH-mutant &1p19q non-co-deleted LGG | 8 | | 2 | 80.0 |  |
| Unknown | 31 | | 9 | 77.5 |  |
| IDH-wildtype GBM | 55 | | 69 | 44.4 | 0.86 |
| IDH-mutant GBM | 7 | | 8 | 46.7 |  |
| Unknown | 2 | | 3 | 0.4 |  |
| **MGMT promoter:** |  | |  |  |  |
| Methylated | 40 | | 18 | 69.0 | 0.25 |
| Unmethylated | 18 | | 14 | 56.3 |  |
| Unknown | 72 | | 64 | 52.9 |  |
| **TERT promoter** |  | |  |  |  |
| Mutant | 57 | | 45 | 55.9 | 0.59 |
| Wildtype | 41 | | 38 | 51.9 |  |
| Unknown | 32 | | 13 | 71.1 |  |
| **Postoperative first-line therapy:** |  | |  |  |  |
| Radiotherapy | 28 | | 19 | 59.6 | 0.39 |
| Chemotherapy | 6 | | 2 | 75.0 |  |
| Chemo-radiotherapy | 74 | | 51 | 59.2 |  |
| No tumor-specific therapy | 22 | | 24 | 47.8 |  |
| **Median age at diagnosis, years (range):** | | 48 (18-80) | | |  |
| **Median follow-up time, months (range):** 29.3 (3.2-82.7) | | | | |  |
| **Median overall survival months (range) in LGG:** 25.3 (6.2-81.3) | | | | |  |
| **Median overall survival months (range) in GBM:** 16.1 (2.7-72.1) | | | | |  |

FPTO: frontal, parietal, temporal or occipital lobe, IDH: isocitrate dehydrogenase, TERT: telomerase reverse transcriptase, MGMT: O6-methylguanine-DNA methyltransferase, GBM: glioblastoma, LGG: Lower grade glioma (Grade II/III glioma). IDH mutation was defined as IDH1-R132H or IDH2-R172K. TERT promoter mutation was defined as TERT-C250T or TERT-C228T. IHC: immunohistochemistry. * Unknown cases were excluded for statistical analyses.
