# Supplementary material for: SCG3 Protein Expression in Glioma Associates With less Malignancy and Favorable Clinical Outcomes
Source: Pathol Oncol Res. 2021 Feb 26;27:594931. doi: 10.3389/pore.2021.594931 (PMC8262226; doi:10.3389/pore.2021.594931)
Supplement: Supplementary file 5 [file Table5.DOCX]

**Supplemental Table 2: Characteristics of the patients included in the Quantitative real-time polymerase chain reaction study**

| **Variables** | **Number of cases**  **(Number of cases in IHC study)** | | **Positive proportion**  **in IHC study, %** | ***p* value** |
| --- | --- | --- | --- | --- |
| **Gender:** |  | |  |  |
| Male | 29 (24) | | 62.5 | 0.728 |
| Female | 17 (15) | | 73.3 |  |
| **Tumor locations:** |  |  |  |  |
| FPTO | 41 (36) | | 63.9 |  |
| Middle-line | 4 (2) | | 100.0 | 0.538 |
| Multiple | 1 (1) | | 100.0 |  |
| **Pathological grade (WHO):** |  |  |  |  |
| II | 8 (7) | | 85.7 |  |
| III | 17 (12) | | 100.0 | 0.001 |
| IV | 21 (20) | | 40.0 |  |
| **Histopathology:** |  | |  | 0.004 |
| Astrocytoma | 1 (1) | | 100.0 |  |
| Oligodendroglioma | 6 (2) | | 100.0 |  |
| Oligoastrocytoma | 18 (16) | | 93.8 |  |
| GBM | 21 (20) | | 40.0 |  |
| **Molecular Subtype:** |  | |  |  |
| IDH-wild-type LGG | 8 (4) | | 100.0 |  |
| IDH-mutant &1p19q co-deleted LGG | 12 (10) | | 80.0 | 0.228 |
| IDH-mutant &1p19q non-co-deleted LGG | 5 (5) | | 100.0 |  |
| IDH-wild-type GBM | 16 (15) | | 40.0 | 1 |
| IDH-mutant GBM | 5 (5) | | 40.0 |  |
| **Median age at diagnosis, years (range):** | 48 (23-69) | | | |

FPTO: frontal, parietal, temporal or occipital lobe, IDH: isocitrate dehydrogenase, GBM: glioblastoma, LGG: Lower grade glioma (Grade II/III glioma). IDH mutation was defined as IDH1-R132H or IDH2-R172K. IHC: immunohistochemistry.
