# Supplementary material for: SCG3 Protein Expression in Glioma Associates With less Malignancy and Favorable Clinical Outcomes
Source: Pathol Oncol Res. 2021 Feb 26;27:594931. doi: 10.3389/pore.2021.594931 (PMC8262226; doi:10.3389/pore.2021.594931)
Supplement: Supplementary file 6 [file Table6.DOCX]

**Supplemental Table 3: Characteristics of the patients included in the Western blot study**

| **Variables** | **Number of cases**  **(Number of cases in IHC study)** | **Positive proportion**  **in IHC study, %** | | ***p* value** |
| --- | --- | --- | --- | --- |
| **Gender:** |  |  | |  |
| Male | 34 (28) | 57.1 | | 0.528 |
| Female | 19 (17) | 70.6 | |  |
| **Tumor locations:** |  | |  |  |
| FPTO | 50 (42) | 59.5 | |  |
| Middle-line | 2 (2) | 100.0 | | 0.515 |
| Multiple | 1 (1) | 100.0 | |  |
| **Pathological grade (WHO):** |  | |  |  |
| II | 12 (7) | 85.7 | |  |
| III | 13 (12) | 100.0 | | 0.001 |
| IV | 28 (26) | 38.5 | |  |
| **Histopathology:** |  |  | |  |
| Astrocytoma | 2 (1) | 100.0 | | 0.002 |
| Oligodendroglioma | 6 (2) | 100.0 | |  |
| Oligoastrocytoma | 17 (16) | 93.8 | |  |
| GBM | 28 (26) | 38.5 | |  |
| **Molecular Subtype:** |  |  | |  |
| IDH-wild-type LGG | 7 (4) | 100.0 | |  |
| IDH-mutant &1p19q co-deleted LGG | 12 (10) | 80.0 | | 0.228 |
| IDH-mutant &1p19q non-co-deleted LGG | 6 (5) | 100.0 | |  |
| IDH-wild-type GBM | 19 (15) | 93.3 | | 1 |
| IDH-mutant GBM | 9 (4) | 100.0 | |  |
| **Median age at diagnosis, years (range):** | 47 (23-69) | | | |

FPTO: frontal, parietal, temporal or occipital lobe, IDH: isocitrate dehydrogenase, GBM: glioblastoma, LGG: Lower grade glioma (Grade II/III glioma). IDH mutation was defined as IDH1-R132H or IDH2-R172K. IHC: immunohistochemistry.
